# Urine Neutrophil Gelatinase‐Associated Lipocalin in Non‐Associative Immune Mediated Hemolytic Anemia: A Prospective Controlled Study in 22 Dogs

**DOI:** 10.1111/jvim.70002

**Published:** 2025-01-27

**Authors:** Vasiliki Lantzaki, Emily A. Fulton, Mark McLaughlin, Euan D. Bennet, Elizabeth A. Conway, Alison E. Ridyard

**Affiliations:** ^1^ University of Glasgow Glasgow UK; ^2^ Paragon Veterinary Referrals Wakefield UK

**Keywords:** AKI, clinical pathology, hematology, renal biomarkers, UNCR

## Abstract

**Background:**

Urine neutrophil gelatinase‐associated lipocalin (uNGAL) is a biomarker for the early diagnosis of AKI.

**Objectives:**

To evaluate uNGAL in dogs with non‐associative immune mediated hemolytic anemia (IMHA) and to evaluate whether uNGAL correlates with disease severity markers, negative prognostic indicators and outcome.

**Animals:**

Twenty‐two dogs with non‐associative IMHA and 14 healthy dogs.

**Methods:**

Prospective case–control study. uNGAL was measured by a commercially available ELISA‐kit and corrected to urine creatinine (uNGAL to creatinine ratio [UNCR]). uNGAL and UNCR of IMHA cases were compared to that of healthy dogs and the correlation with other clinicopathological markers was evaluated. uNGAL and UNCR were also compared between dogs with a CHAOS or ASA score < 3 and ≥ 3.

**Results:**

uNGAL and UNCR were significantly higher in dogs with IMHA when compared to healthy controls (uNGAL median 114.58 and 0.43 ng/mL, respectively, *p* < 0.001; UNCR median 174.87 and 0.13 ng/mg, respectively, *p* < 0.001). uNGAL and UNCR were moderately positively correlated with urea (*p* = 0.005, *r* = 0.58, 0.20–0.81 95% CI and *p* = 0.001, *r* = 0.64, 0.29–0.84 95% CI, respectively) and total bilirubin (*p* = 0.003, *r* = 0.60, 0.22–0.82 95% CI and *p* = 0.002, *r* = 0.62, 0.25–0.83 95% CI, respectively). These were also significantly higher in dogs with hemoglobinuria compared to those without (uNGAL: median 269 and 30.99 ng/mL, respectively, *p* < 0.001; UNCR: median 585.3 and 352 37.47 ng/mg, respectively, *p* < 0.001). There was no statistically significant difference in uNGAL or UNCR when assessing survival to discharge (*p* = 0.24 and *p* = 0.16, respectively, 95% CI).

**Conclusions:**

This study suggests that renal injury might be underappreciated in dogs with IMHA.

**Abbreviations**
AKI: acute kidney injury
ALP: alkaline phosphatase
ALT: alanine transaminase
ASA: American Society of Anesthesiologists
CHAOS: canine hemolytic anemia objective score
CRP: C‐reactive protein
HCT: hematocrit
hpf: high power field
IMHA: immune mediated hemolytic anemia
NGAL: neutrophil gelatinase‐associated lipocalin
PLT: platelet
RBC: red blood cell
UNCR: urine neutrophil gelatinase‐associated lipocalin to creatinine ratio
UPC: urine protein to creatinine ratio
USG: urine specific gravity
WBC: white blood cells

## 1 Introduction

Immune mediated hemolytic anemia (IMHA) is a prevalent disease in dogs [1, 2, 3] and is characterized by the production of anti‐red blood cell (RBC) antibodies which attach to the RBC membrane and act as opsonins. This leads to activation of the complement cascade and subsequent intravascular hemolysis or to extravascular hemolysis by the mononuclear phagocyte system [4]. IMHA carries a guarded prognosis and despite advances in treatment, fatality remains high, with the most recent studies citing a case fatality rate of 30%–50% [5, 6, 7]. While age, high serum bilirubin, alkaline phosphatase (ALP), alanine transaminase (ALT), and urea concentrations above the reference range, hypoproteinemia, thrombocytopenia, autoagglutination, and prolonged clotting times have all been associated with a worse outcome, only urea and bilirubin are consistently reported as negative prognostic indicators [1, 2, 5, 8, 9, 10, 11].

It is speculated that abnormally high plasma urea concentration in IMHA results, at least in part, from hemoglobin‐induced AKI [12, 13, 14, 15], hypoxia‐induced renal injury [16], and concurrent thromboembolic complications [2, 3, 4, 5, 6, 7, 8, 9, 10, 11, 12, 13, 14, 15, 16]. Pre‐renal factors, such as gastrointestinal bleeding might also contribute [17, 18, 19]. However, the incidence of AKI in dogs with IMHA is currently unknown possibly due to the fact that a diagnosis of AKI has historically been based on serum creatinine and other insensitive markers of AKI such as the presence of casts on urine sediment examination [20, 21, 22, 23]. AKI is a potentially fatal complication of various diseases and has been associated with a worse prognosis for hospitalized animals irrespective of the underlying cause [21, 24] and it might therefore have prognostic relevance in IMHA.

Neutrophil gelatinase‐associated lipocalin (NGAL) is a 25 kDa protein belonging to the lipocalin family [25]. Urine NGAL (uNGAL) is an early diagnostic marker for AKI in dogs that correctly detects non‐azotaemic AKI (IRIS grade I AKI) in both experimentally induced kidney injury [26, 27, 28, 29, 30, 31, 32] and clinical cases [15, 33, 34, 35, 36].

The aim of this study was to evaluate uNGAL as a marker of renal tubular injury in dogs with non‐associative IMHA. A secondary aim was to see whether uNGAL and uNGAL to creatinine ratio (UNCR) correlated with other negative prognostic indicators and was predictive of outcome. We hypothesized that both uNGAL and UNCR would be higher in dogs with IMHA when compared to a cohort of healthy dogs and that abnormally high uNGAL and UNCR would be associated with more severe disease and worse outcome.

## 2 Materials and Methods

### 2.1 Study Design

This was a prospective case–control study evaluating a group of dogs with non‐associative IMHA and comparing them to a cohort of healthy dogs.

### 2.2 Study Cohort

Client‐owned dogs presenting to the University of Glasgow Small Animal Hospital from April 2017 to August 2023 and diagnosed with naturally occurring non‐associative IMHA were included in this study. The ACVIM consensus statement criteria for the diagnosis of IMHA were used to establish diagnosis [37]. Dogs were included if they had a confirmed diagnosis of IMHA based on the presence of anemia and two of three of the following markers of immune mediated red cell destruction: (1) positive in‐saline agglutination, (2) positive direct Coombs test (3) moderate‐marked spherocytosis (diagnostic of IMHA). Alternatively, where there was only one marker of immune‐mediated red cell destruction, dogs were included if they also had one or more markers of hemolysis (hyperbilirubinemia, bilirubinuria, hemoglobinuria, hemoglobinemia, erythrocyte ghosts; supportive diagnosis of IMHA). To assess for evidence of a putative trigger, anamnestic questions were asked, including date of last vaccination, recent medications, exposure to fleas and ticks, flea and tick prevention used and travel history; routine assessment for the presence of infectious agents was not performed in the absence of history of travel to an endemic region. In addition to routine blood work (complete blood count and serum biochemistry), imaging, including thoracic radiographs and abdominal ultrasound were performed to assess for the presence of an inflammatory or neoplastic focus. Dogs with a putative trigger (associative IMHA) were excluded from the study as were dogs that had received steroids for more than 48 h prior to the presentation to our hospital. Dogs were also excluded if there was no urinalysis performed on presentation or if there was evidence of pyuria (> 5 white blood cells [WBC]/high power field [hpf]) [38].

The signalment, physical examination findings, and selected clinicopathological data were recorded for each case. This included disease severity markers such as hematocrit value (HCT) and presence of hemoglobinuria and previously identified negative prognostic indicators such as slide agglutination results, platelet count, serum creatinine, urea, total bilirubin, ALT, ALP, albumin, and total protein. Other clinicopathological data included blood smear interpretation, direct Coombs test, white blood cell count, neutrophil count and c‐reactive protein (CRP)^1^ ^,^ ^2^. Data from serial evaluation of serum biochemistry during initial hospitalization was recorded when available. Leukocytosis was defined as WBC > 12 × 10^9^/L, neutrophilia as neutrophil count > 11.8 × 10^9^/L, thrombocytopenia as platelet count < 200 × 10^9^/L, hyperbilirubinemia as the presence of total bilirubin > 1.17 mg/dL, and high urea when urea > 50.5 mg/dL, using the reference interval of our reference laboratory. Where possible, AKI was diagnosed and graded based on the International Renal Interest Society (IRIS) guidelines [39]. Urine specific gravity (USG), presence of pyuria, presence of hemoglobinuria, presence of glucosuria, proteinuria (defined as urine protein to creatinine ratio [UPC] > 0.5) and creatinine concentration were recorded from the urinalysis performed at the time of presentation.^3^ ^,^ ^4^

The treatments and number of transfusions were recorded; all therapeutic decisions were made independently by the primary clinician. The Canine Hemolytic Anemia Objective Score (CHAOS) and American Society of Anesthesiologists score (ASA) were calculated retrospectively based on the previously described criteria (Tables S1–S3) [5, 40]. Survival to discharge, 30, 60, and 90 days from admission was recorded when available. Records were also retrospectively assessed for clinical suspicion of thromboembolic event during the period of hospitalization. This included confirmation of presence of thrombi as seen on imaging, report of unexplained tachypnea raising the suspicion of pulmonary thromboembolism (PTE), or report of signs of neurological disease suspicious for central nervous system (CNS) thromboembolism.

### 2.3 Control Cohort

Our control cohort comprised 14 healthy staff or student owned dogs. Dogs were deemed to be healthy based on history, a health questionnaire, clinical examination, and urinalysis. They were excluded if they had been on any long‐term medications other than routine endo‐ and ectoparasiticides.

### 2.4 Sample Collection

Mid‐stream voided urine samples were collected, unless cystocentesis was clinically required, at the time of presentation from all dogs in the study cohort. Voided urine samples were collected from the healthy dogs within 24 h of health screening. Urine samples were divided into 1 mL aliquots and frozen at −20°C within 24 h of collection, then transferred to −80°C storage within a week from collection until batch analysis at a later date. The maximum duration of storage in this study was 6 years.

### 2.5 uNGAL ELISA Protocol

uNGAL concentrations were measured using a dog‐specific sandwich enzyme‐linked immunosorbent assay (ELISA; Bioporto, Gentofte, Denmark)^1^, which is validated for use in dogs [41].^5^ Aliquots of urine from all cases (IMHA and healthy controls) were thawed and prepared as per manufacturer instructions. Briefly, samples were centrifuged on a low speed to remove potential particulate matter, then 10 μL was diluted with diluent to create a 1:100 dilution. Additional dilutions (1:10 or 1:20 dilution) were required for some samples. The protocol was followed as per the manufacturer instructions with all samples assayed in duplicate. The plate was read with an ELISA reader at 450 nm (reference wavelength 650 nm). uNGAL concentration was expressed as an absolute uNGAL value (ng/ml) and the UNCR calculated and expressed in ng/mg. Urine creatinine concentration was measured by ultraviolet–visible absorbance spectrophotometry (Cecil ce373 spectrophotometer) as part of routine urinalysis. Any case that had an unmeasurably low uNGAL was reported as 0.2 ng/mL [35, 42].

### 2.6 Statistical Analysis

Nonparametric tests were used for all analyses. Baseline clinical characteristics were summarized, and medians and range reported. The uNGAL and UNCR values of IMHA cases were compared to that of healthy dogs using the Mann–Whitney U test. uNGAL and UNCR were also compared to that of healthy dogs from previous studies; 0.04–12.9 ng/mL and 0.0–1.57 ng/mg for uNGAL and UNCR, respectively [35, 36, 43, 44]. Spearman's correlation was used to evaluate the correlation between uNGAL and UNCR and continuous data, which included HCT, platelet count, urea, creatinine, total bilirubin, ALT, ALP, total proteins, albumin, and CRP. Mann–Whitney U test was used for comparison of uNGAL and UNCR between dogs with a CHAOS or ASA score < 3 or ≥ 3, categorical disease severity markers (transfusion requirements, presence of hemoglobinuria, suspected thromboembolism) and outcome at discharge, 30, 60, and 90 days. For all analysis, *p* < 0.05 was considered to be statistically significant. Analyses were performed using GraphPad Prism version 10.1.0 statistical software.^6^

## 3 Results

### 3.1 Study Cohort Characteristics

Forty‐two dogs with suspected non‐associative IMHA were initially recruited. Of these, three dogs were excluded; one was subsequently diagnosed with phosphofructokinase deficiency, and two were diagnosed with associative IMHA, secondary to neoplasia and clindamycin administration, respectively. Another 17 dogs were excluded as urine samples were not collected or analyzed at the time of presentation. Twenty‐two dogs were included in the final analysis, sixteen fulfilling the ACVIM consensus statement diagnostic criteria for IMHA, and six the supportive criteria [37] (Table S1). The dogs ranged in age from 1 to 12 years, with a median of 8 years. There were 4 male (1 neutered, 3 entire) and 18 female (13 neutered and 5 entire) dogs. Breeds included six Cocker spaniel, three springer spaniels, two crossbreed dogs, two Schnauzers, two Shit‐tzu and one each of Golden Retriever, Labrador Retriever, German Shepherd, Whippet, Cairn Terrier, Irish Setter, Jack Russel Terrier.

Fourteen healthy dogs were included in the study. The median age was 6 years (range 1 to 11). The group consisted of nine male dogs (seven neutered, two entire) and five female neutered dogs. Breeds included two Border Collie, two English springer spaniel, and one each of Wheaten terrier, Golden retriever, Chihuahua, Labrador retriever, Standard schnauzer, Dachshund, Vizsla, Rottweiler, Flat Coated retriever and crossbreed.

### 3.2 Clinicopathological Data

Complete blood count (CBC) was available in all 22 dogs. The HCT ranged from 4.7%–25.5% (median 16.1%). Leukocytosis was noted in 20 of 22 dogs (90.9%; WBC median 18.5 × 10^9^/L, range 8.1–48.9 × 10^9^/L) with neutrophilia present in 15 of 22 dogs (68.2%; neutrophil count median 14.14 × 10^9^/L, range 6–29.67 × 10^9^/L). A 11 of 22 (50%) were thrombocytopenic (platelet count median 203 × 10^9^/L, range 81–750 × 10^9^/L).

Serum biochemistry was available in all 22 dogs. Serum urea concentration above the reference range was present in 8 of 22 dogs (36%; median 47 mg/dL, range 25–162 mg/dL) with creatinine concentration above reference range in one dog (5%; median 0.67 mg/dL, range 0.32–2.86 mg/dL). Hyperbilirubinemia was noted in 17/22 (77%; median 1.40 mg/dL, range 0.23–39 mg/dL). CRP concentration was available in 13/22 dogs, ranging from 9 to 348.1 mg/L (median 164.3 mg/L). Repeated serum biochemistry was performed during the initial hospitalization in 13 of 22 dogs; of these, 1 developed IRIS grade I AKI.

The median USG was 1.030 (range 1.017–1.050, *n* = 22). The median UPC ratio was 0.76 (range 0.05–16.5, *n* = 21) with 12 dogs being proteinuric (UPC > 0.5). One case was excluded due to a repeatable UPC ratio measurement of 314 which was considered to be erroneous due to analytical interference. Glucose was present on urine dipstick in 1 of 22 dogs (100 mg/dL). Hemoglobinuria was present in 11 of 22 dogs (50%). Selected clinicopathological data are included in Table 1 and Tables S2 and S3.

**TABLE 1:** Clinicopathological information of IMHA cases relating to IMHA diagnosis and renal injury/function.

IMHA diagnosis information		Renal injury‐function information	
	Number of dogs		Median (range)
Slide agglutination (positive/negative)	20/2	Serum creatinine (mg/dl)	0.67 (0.32–2.86)
Spherocytosis (positive/negative/equivocal)	16/4/2	Serum urea (mg/dl)	47 (25–162)
Direct Coombs (positive/negative/not assessed)	16/0/6	USG	1.030 (1.017–1.050)
Hemoglobinuria (positive/negative)	11/11	UPC	0.76 (0.05–16.5)
Hyperbilirubinemia (Tbil > 0.58 mg/dL) (present/non‐present)	17/5	uNGAL (ng/ml)	114.58 (0.2–1166.77)
ACVIM consensus statement diagnosis category (diagnostic/supportive)	16/6	UNCR (ng/mg)	174.87 (0.15–1035.1)

Abbreviations: IMHA: immune‐mediated hemolytic anemia, Tbil: Total bilirubin, UNCR: uNGAL to creatinine ratio, uNGAL: Urine neutrophil gelatinase‐associated lipocalin.

### 3.3 Treatment and Survival Data

Two dogs were treated with a single immunosuppressive agent, 18 dogs with 2 agents and 2 dogs with 3 agents. All dogs received glucocorticoids; 19 were treated with dexamethasone^7^ (IV, median dosage 0.3 mg/kg/d; range, 0.07–0.6 mg/kg/day), with 12 of them followed by prednisolone^8^ (PO, median dose 1.79 mg/kg/day; range 0.91–2.1 mg/kg/day), while the remaining 3 dogs received prednisolone^9^ only (PO, median dose 2 mg/kg/day; range 1.94–2.02 mg/kg/day). Twenty dogs received a second line immunosuppressive drug during initial hospitalization; mycophenolate^10^ (*n* = 17; PO, median dose 19 mg/kg/d; range 7.9–24.2 mg/kg/d), cyclosporine^11^ (*n* = 2) at a median dosage of 6.4 mg/kg/d (PO, range 5.0–7.8 mg/kg/d), or azathioprine^12^ (*n* = 1) at a dose of 1.6 mg/kg/d (PO); 2 dogs also received intravenous human immunoglobulin^13^ (IV, 0.5 g/kg). Clopidogrel^14^ was used for thromboprophylaxis in all dogs, (PO, median dose 2.1 mg/kg/d; range 1–3.8 mg/kg/d) and one dog also received dalteparin^15^ at a dose of 150 units/kg every 8 h (SC). Eighteen dogs (82%) received ≥ 1 packed RBC transfusion (IV). Information on the treatment regimens used in each case is included in Table S4.

A 15 of the 22 dogs (68%) included in this study survived to discharge. Of the seven that did not survive to discharge, four were euthanized, and three died naturally. Of the four that were euthanized, teo dogs were euthanized on Day 1 due to signs of neurological disease attributed to a thromboembolic event, and two were euthanized on Day 6 and 7 because of persistent hemolysis. Four dogs subsequently died, one due to relapse of the IMHA (58 days after initial presentation), two due to unrelated reasons (319‐ and 473‐days after initial presentation) and one due to an unknown reason 68 days after diagnosis. A total of 5 of 22 dogs had a suspected or known thromboembolism; 1 dog was found to have a caudal venal cava thrombus as shown on abdominal ultrasound, 2 dogs had signs of neurological disease suspicious of CNS thrombosis and another 2 dogs had acute development of signs of respiratory disease, unexplained by thoracic radiography and suspicious of PTE. Further survival data are presented in Table S5.

### 3.4 uNGAL Results

**FIGURE 1:**
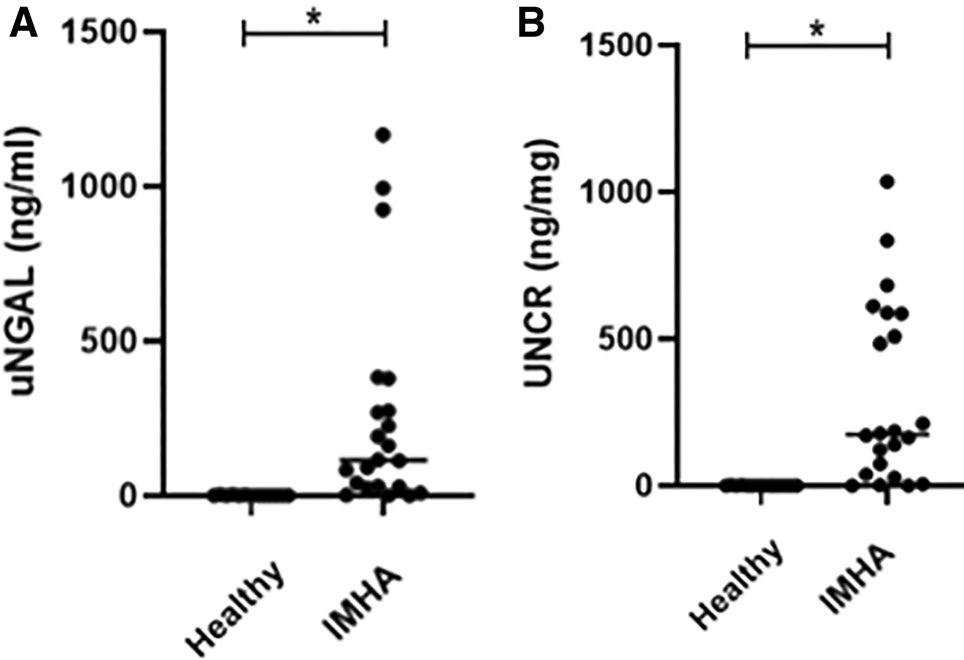
Scatter plots of healthy controls (*n* = 14) and dogs with IMHA (*n* = 22). (A) Urine NGAL in the two groups (*p* < 0.001), (B) UNCR in the two groups (*p* < 0.001). Dots represent individual dogs and line represents median, *indicates a statistically significant difference. IMHA: immune‐mediated hemolytic anemia, UNCR: uNGAL to creatinine ratio, uNGAL: urine neutrophil gelatinase‐associated lipocalin.

uNGAL concentration ranged between 0.2 and 1166.77 ng/mL (median 114.58 ng/mL) in the IMHA group while in the healthy control group, uNGAL concentration ranged between 0.2 ng/mL and 3.51 ng/mL (median 0.43 ng/mL). There was a statistically significant difference between the 2 groups, with the IMHA dogs having significantly higher uNGAL concentration compared to healthy dogs (*p* < 0.001; Figure 1A). Nineteen dogs (86%) with IMHA had uNGAL concentration that exceeded that of the highest value in the healthy control group.

UNCR was calculated for all dogs and it ranged between 0.15 and 1035.1 ng/mg (median 174.87 ng/mg) in dogs with IMHA and from 0.2 to 1.67 ng/mg (median 0.13 ng/mg) in the healthy group. The difference between the 2 groups was statistically significant (*p* < 0.001; Figure 1B). Twenty IMHA dogs (91%) had a UNCR exceeding that of the highest value in the healthy control group.

When uNGAL and UNCR results were compared to reported normal ranges [35, 36, 43, 44] similar results were obtained; 18 of 22 dogs (82%) had high uNGAL concentration (uNGAL > 12.9 ng/mL) and 20 of 22 dogs (91%) had high UNCR results (UNCR > 1.57 ng/mg).

### 3.5 Correlation Between uNGAL, UNCR, and Continuous Clinicopathological Data

There was a negative, although not statistically significant, relationship between HCT and uNGAL (*p* = 0.27, *r* = −0.25, −0.61 to 0.21 95% CI) and between HCT and UNCR (*p* = 0.19, *r* = −0.29, −0.64 to 0.16 95% CI). There was no correlation between uNGAL and WBC (*p* = 0.44, *r* = 0.17, −0.28 to 0.56 95% CI), neutrophil count (*p* = 0.27, *r* = 0.25, −0.2 to 0.61 95% CI) or platelet count (*p* = 0.12, *r* = 0.34, −0.11 to 0.67 95% CI). Similarly, UNCR was not correlated with either WBC (*p* = 0.36, *r* = 0.21, −0.25 to 0.59 95% CI), neutrophil count (*p* = 0.20, *r* = 0.29, −0.17 to 0.64 95% CI) or platelet count (*p* = 0.20, *r* = 0.28, −0.17 to 0.64 95% CI).

**FIGURE 2:**
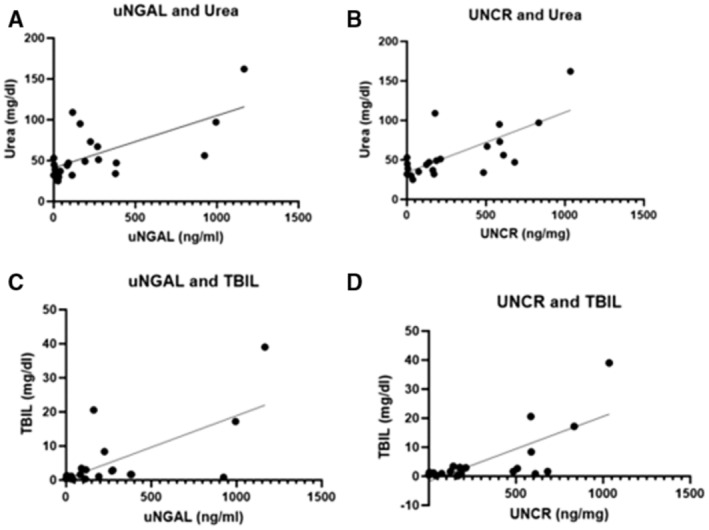
Scatter plots of Spearman's correlation results from dogs with IMHA (*n* = 22). Dots represent individual dogs, *indicates a statistically significant correlation: (A) uNGAL and urea (*p* = 0.005), (B) UNCR and urea (*p* = 0.001), (C) uNGAL and total bilirubin (*p* = 0.003), (D) UNCR and total bilirubin (*p* = 0.002). IMHA: immune‐mediated hemolytic anemia, TBIL: total bilirubin, UNCR: uNGAL to creatinine ratio, uNGAL: urine neutrophil gelatinase‐associated lipocalin.

There was a statistically significant moderate correlation between urine uNGAL and serum urea (*p* = 0.005, *r* = 0.58, 0.20–0.81 95% CI) and uNGAL and total bilirubin (*p* = 0.003, *r* = 0.60, 0.22–0.82 95% CI; Figure 2). Similarly, UNCR was moderately correlated with both serum urea (*p* = 0.001, *r* = 0.64, 0.29–0.84 95% CI) and total bilirubin (*p* = 0.002, *r* = 0.62, 0.25–0.83 95% CI). There was, however, no correlation between uNGAL and serum creatinine (*p* = 0.49, *r* = 0,16, −0.30 to 0.56 95% CI), ALT (*p* = 0.67, *r* = 0,10, −0.37 to 0.53 95% CI), ALP (*p* = 0.31, *r* = 0,23, −0.23 to 0.61 95% CI), total protein (*p* = 0.61, *r* = 0,11, −0.33 to 0.52 95% CI), albumin (*p* = 0.72, *r* = 0,08, −0.36 to 0.50 95% CI) or CRP (*p* = 0.15, *r* = 0,43, −0.18 to 0.80 95% CI) or between UNCR and serum creatinine (*p* = 0.72, *r* = 0,08, −0.36 to 0.50 95% CI), ALT (*p* = 0.50, *r* = 0,16, −0.32 to 0.57 95% CI), ALP (*p* = 0.33, *r* = 0,22, −0.24 to 0.61 95% CI), total protein (*p* = 0.51, *r* = 0,15, −0.30 to 0.55 95% CI), albumin (*p* = 0.56, *r* = 0,13, −0.32 to 0.53 95% CI), or CRP (*p* = 0.19, *r* = 0,39, −0.22 to 0.78 95% CI; Table 2).

**TABLE 2:** Correlation between selected clinicopathological variables and uNGAL and UNCR.

			UNCR			uNGAL		
	Number	Median (range)	*R*	95% confidence interval	*p*	*R*	95% confidence interval	*p*
HCT (%)	*n* = 22	16.1 (4.7–25.5)	−0.29	−0.64 to 0.16	*p* = 0.19	−0.25	−0.61 to 0.21	*p* = 0.27
WBC (10^9^/L)	*n* = 22	18.5 (8.1–48.9)	0.21	−0.25 to 0.59	*p* = 0.36	0.17	−0.28 to 0.56	*p* = 44
Neutrophils (10^9^/L)	*n* = 22	14.14 (6–29.67)	0.29	−0.17 to 0.64	*p* = 0.20	0.25	−0.2 to 0.61	*p* = 0.27
PLT (10^9^/L)	*n* = 22	203 (81–750)	0.28	−0.17 to 0.64	*p* = 0.20	0.34	−0.11 to 0.67	*p* = 0.12
Creatinine (mg/dl)	*n* = 22	0.67 (0.32–2.86)	0.08	−0.36 to 0.50	*p* = 0.72	0.16	−0.30 to 0.56	*p* = 0.49
Urea (mg/dl)	*n* = 22	47 (25–162)	0.64	0.29 to 0.84	*p* = 0.001	0.58	0.20–0.81	*p* = 0.005
Total bilirubin (mg/dl)	*n* = 22	1.4 (0.23–39)	0.62	0.25 to 0.83	*p* = 0.002	0.60	0.22 to 0.82	*p* = 0.003
Total protein (g/l)	*n* = 22	72 (52–93)	0.15	−0.30 to 0.55	*p* = 0.51	0.11	−0.33 to 0.52	*p* = 0.61
Albumin (g/l)	*n* = 22	32.5 (22–42)	0.13	−0.32 to 0.53	*p* = 0.56	0.08	−0.36 to 0.50	*p* = 0.72
ALT (U/l)	*n* = 20	39.5 (14–2704)	0.16	−0.32 to 0.57	*p* = 0.50	0.10	−0.37 to 0.53	*p* = 0.67
ALP (U/l)	*n* = 21	172 (43–578)	0.22	−0.24 to 0.61	*p* = 0.33	0.23	−0.23 to 0.61	*p* = 0.31
CRP (mg/l)	*n* = 13	164.3 (9–348.1)	0.39	−0.22 to 0.78	*p* = 0.19	0.43	−0.18 to 0.80	*p* = 0.15

*Note:* Results are reported as median and range.

Abbreviations: ALP: alkaline phosphatase, ALT: alanine transaminase, HCT: hematocrit value, PLT: platelets, WBC: white blood cells.

### 3.6 uNGAL, UNCR, and Categorical Clinicopathological Data

**FIGURE 3:**
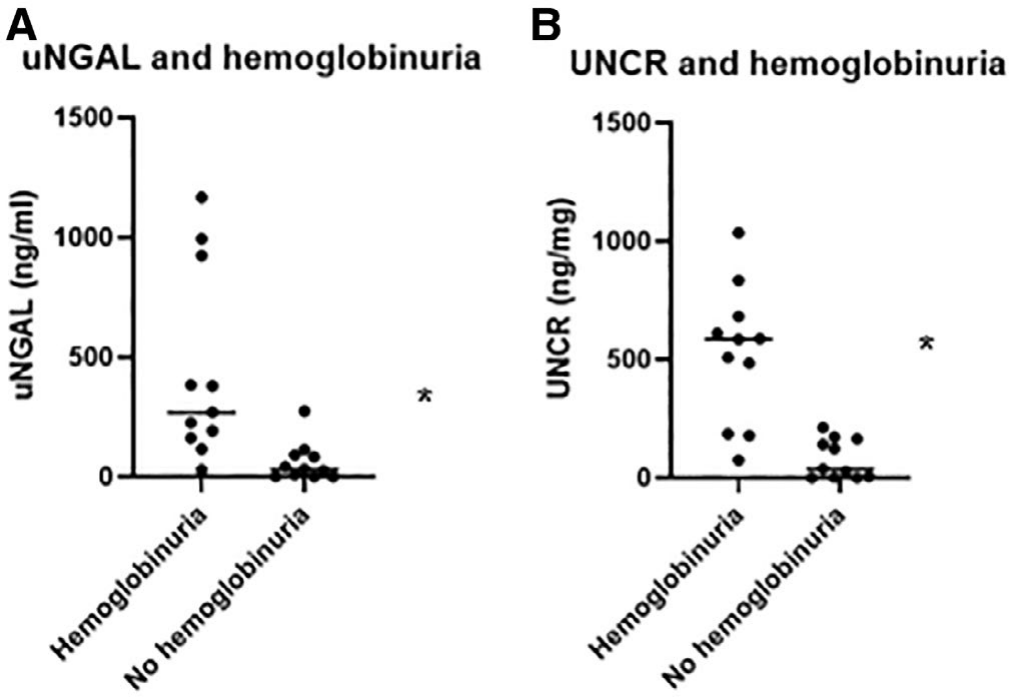
Scatter plot of IMHA cases (*n* = 22). (A) Comparison of uNGAL between dogs with presence or absence of hemoglobinuria (*p* < 0.001). (B) Comparison of UNCR between dogs with presence or absence of hemoglobinuria (*p* < 0.001). Dots represent individual dogs. Line represents median, *indicates a statistically significant difference. IMHA: immune‐mediated hemolytic anemia, UNCR: uNGAL to creatinine ratio, uNGAL: urine neutrophil gelatinase‐associated lipocalin.

A statically significant difference in uNGAL values was noted when dogs with and without hemoglobinuria were compared (median 269 and 30.99 ng/mL, respectively, *p* < 0.001). UNCR was also found to be statistically significantly different when comparing dogs with and without hemoglobinuria (median 585.3 and 37.47 ng/mg, respectively, *p* < 0.001; Figure 3). There was no difference in uNGAL or UNCR when dogs with a positive or negative slide agglutination test were compared (*p* = 0.08 for both), although only a small number of dogs (*n* = 2) had negative slide agglutination test in this cohort (Table 3).

**TABLE 3:** Results of comparison of uNGAL and UNCR between selected categorical data.

			UNCR		uNGAL	
	Total number	Number (pos/neg)	Median pos /median neg (ng/mg)	*p*	Median pos /median neg (ng/ml)	*p*
Slide agglutination	*n* = 22	20/2	182.3/3.46	*p* = 0.08	139.1/5.1	*p* = 0.08
Hemoglobinuria	*n* = 22	11/11	585.3/37.47	*p* < 0.001	269/30.99	*p* < 0.001

Abbreviations: neg: negative, pos: positive, UNCR: urine neutrophil gelatinase‐associated lipocalin to creatinine ratio.

### 3.7 uNGAL, UNCR, and Disease Severity

**FIGURE 4:**
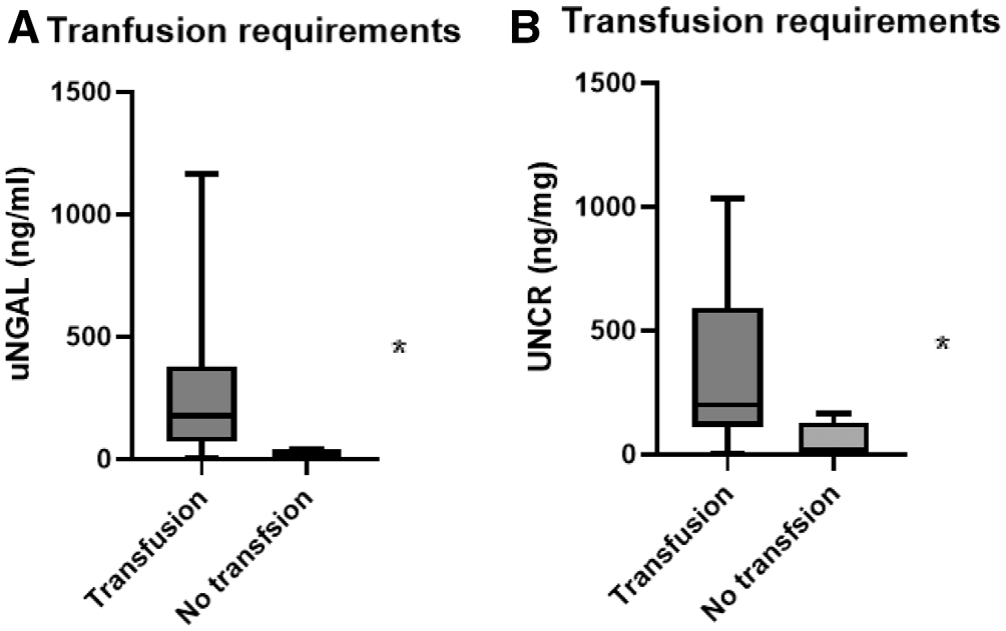
Box and whiskers of IMHA cases (*n* = 22) grouped depending on transfusion requirements. (A) uNGAL in dogs that required at least one transfusion versus those that received no transfusion (*p* = 0.015). (B) UNCR in dogs that required at least one transfusion versus those that received no transfusion (*p* = 0.015). Box includes the interquartile range, and the line represents the median. The whiskers represent the range, *indicates a statistically significant difference. IMHA: immune‐mediated hemolytic anemia, UNCR: uNGAL to creatinine ratio, uNGAL: urine neutrophil gelatinase‐associated lipocalin.

Dogs that required a transfusion had a significantly higher uNGAL concentration (median 177.3 ng/mL) when compared to those that did not (median 15.7 ng/mL; *p* = 0.015). Similarly, a significantly higher UNCR was observed in dogs that required transfusion than those that did not (median 199.3 and 14.03 ng/mg, respectively, *p* = 0.015; Figure 4).

There was no significant difference in the uNGAL or UNCR results of dogs with suspect thromboembolism when compared to that of dogs with no suspicion of thromboembolism (*p* = 0.4 and *p* = 0.25, respectively). There was no difference in uNGAL or UNCR results when comparing dogs with a CHAOS score < 3 and ≥ 3, (*p* = 0.70 and *p* = 0.94, respectively), nor when dogs with CHAOS score < 5 were compared with dogs with a CHAOS score ≥ 5 (*p* = 0.14 and *p* = 0.052, respectively). uNGAL and UNCR results did not significantly differ between ASA < 3 and ≥ 3 (*p* = 0.31 and *p* = 0.26, respectively).

### 3.8 uNGAL, UNCR, and Outcome

**FIGURE 5:**
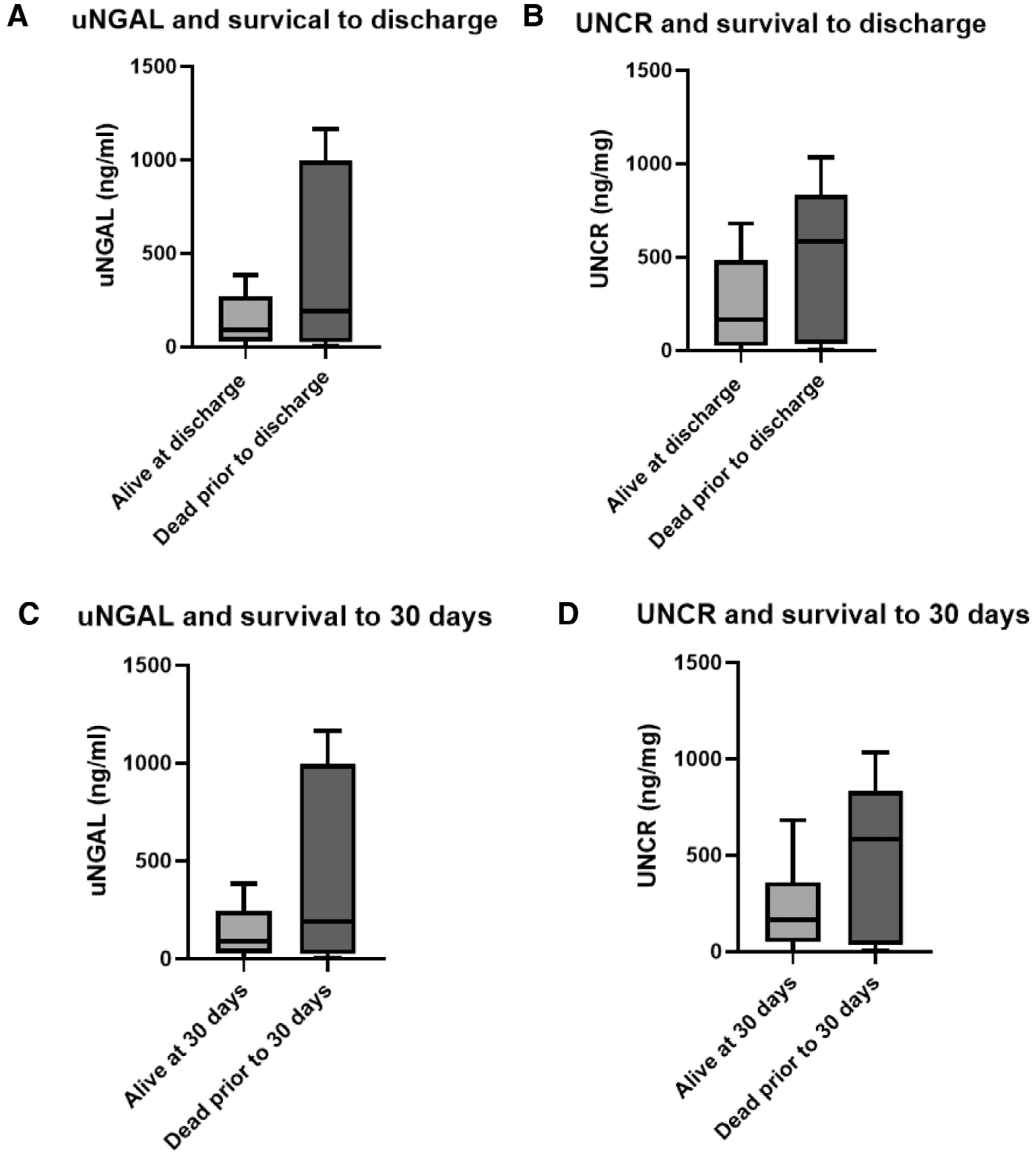
Box and whiskers of baseline uNGAL and UNCR levels in IMHA cases and survival: (A) uNGAL and survival to discharge (*p* = 0.24), (B) UNCR and survival to discharge (*p* = 0.16), (C) uNGAL and survival to 30 days (*p* = 0.27). (D) UNCR and survival to 30 days (*p* = 0.21). Box includes the interquartile range, and the line represents the median. The whiskers represent the range. IMHA: immune‐mediated hemolytic anemia, UNCR: uNGAL to creatinine ratio, uNGAL: urine neutrophil gelatinase‐associated lipocalin.

There were no differences in uNGAL and UNCR results when comparing dogs that survived to discharge with those that did not (*p* = 0.24 and *p* = 0.16, respectively 95% CI). uNGAL concentrations were not statistically different between dogs that survived to 30, 60, or 90 days, and those that did not (*p* = 0.27, *p* = 0.24, and *p* = 0.28 respectively). Similarly, there was no statistical difference in the UNCR results when comparing dogs that survived to 30, 60, or 90 days, with those that did not (*p* = 0.21, *p* = 0.13, and *p* = 0.16 respectively; Figure 5).

## 4 Discussion

In this study we evaluated uNGAL levels in dogs with IMHA as a sensitive marker of renal tubular injury and thus AKI. We found that uNGAL (and UNCR) was abnormally high in the majority of dogs with IMHA, and observed moderate positive correlations with previously identified negative prognostic indicators, urea, and bilirubin.

uNGAL was first shown to be an early marker of AKI in an experimental model of ischemic renal injury in mice, with urinary concentrations correlating with the degree and duration of renal ischemia [45]. Subsequent clinical studies in human medicine [46, 47, 48, 49, 50], corroborated this observation, with uNGAL increasing at least 24 h prior to an increase in serum creatinine. There is a correlation between uNGAL and severity of tubulointerstitial injury in humans with IgA nephropathy, chronic kidney disease, and AKI [51, 52, 53]. In dogs, uNGAL is abnormally high in both experimentally‐induced [26, 27, 28, 29, 30, 31, 32] and naturally occurring AKI [15, 33, 34, 36]. It has been previously shown that UNCR predicted the development of azotaemic AKI by a median of 8 days earlier than creatinine in gentamicin‐induced AKI in dogs [31]. The ability of uNGAL to correctly detect non‐azotemic AKI (IRIS grade I AKI) has also been demonstrated in clinical cases of AKI [34, 36, 54]. Finally, similar to human medicine and other experimental models, a correlation between uNGAL and severity of kidney lesions was seen in gentamicin‐induced AKI in dogs [29]. Our findings of abnormally high uNGAL and UNCR in the majority of dogs with IMHA therefore indicate that renal tubular injury might be common in dogs with IMHA.

In this study we elected to report both uNGAL and UNCR as it is currently unclear which is more appropriate to assess for the presence of AKI; although correction to creatinine (UNCR) is common practice to avoid fluctuations associated with changes in urine concentration, the validity of using this methodology during AKI has been questioned, as urine creatinine concentrations fluctuate and there is potential for overestimation of tubular damage when UNCR, as opposed to uNGAL is used [55].

Renal injury and the development of AKI in dogs with IMHA is believed to be multifactorial. Tissue hypoxia due to anemia has been suggested to play an important role in the development of AKI in IMHA [16]. More specifically a HCT of < 10% has previously been reported to severely impair tissue oxygenation [56, 57] and the resultant tissue damage leads to the release of chemotaxins that orchestrate an inflammatory response. Although a statistically significant association between UNCR and HCT value was not seen in our cohort, uNGAL and UNCR were significantly different between dogs that required a transfusion and those that did not, supporting the potential role of hypoxia.

Hemoglobinuria is associated with the development of AKI in dogs [12, 14, 15]. The proposed mechanisms include enhanced 4‐hydroxynonenal reactivity in the presence of free heme leading to tubular injury [14], precipitation of free hemoglobin in the acidic ultrafiltrate leading to cast formation that obstruct the tubular lumen [58, 59] and finally reduced bioavailability of nitric oxide due to reaction with hemoglobin with a resultant impairment in tissue perfusion [60]. In our study, uNGAL and UNCR were found to be significantly higher in dogs with hemoglobinuria compared to those without, which might further support a cause‐effect relationship between hemoglobinuria and renal tubular injury. However, this observation could be due to interference between hemoglobin and the NGAL assay. The specificity of uNGAL in the presence of hematuria has been questioned with one study reporting a mild correlation between hematuria and uNGAL (*R* ^2^ = 0.21), indicating that blood might interfere with uNGAL measurement even in the absence of overt kidney injury evidenced as assessed by serum creatinine concentration rise [61]. However, there is negligible interference of hemoglobinuria with uNGAL after experimental addition of hemoglobin to the urine of healthy dogs, suggesting that the presence of hemoglobinuria does not significantly affect uNGAL concentration measurement [62]. Further evaluation is clearly needed.

Thromboembolism has also been suggested as an etiological factor leading to AKI in dogs with IMHA [16]. Carr et al. reported thromboembolism in 80% of the dogs with IMHA that had postmortem examination, with 32% of these dogs having emboli involving the kidneys [8]. Thromboembolism further disturbs tissue perfusion, aggravating progressive hypoxic tissue necrosis. In our study 5 of 22 dogs had a known or suspected thromboembolism. Although no significant difference was found in uNGAL or UNCR of dogs with a known/suspected thromboembolic event as compared to that of dogs with no evidence of thromboembolism in our cohort, histopathology was not performed and therefore unidentified thromboemboli cannot be excluded as the cause of abnormally high uNGAL in our cohort.

In this cohort, while the majority of dogs had abnormally high uNGAL and UNCR, only 1 dog had creatinine above the reference range at baseline and of the 13 dogs that had serial evaluation of serum biochemistry during the initial hospitalization only 1 progressed to non‐azotaemic AKI. This observation is not unexpected, as while uNGAL is a highly sensitive marker of renal tubular damage, tubular injury might be transient, and doesn't necessarily result in a measurable functional change, particularly if the cause of the renal injury/insult has been resolved. For this reason, the use of clinically applicable AKI phenotype matrix‐based systems of classification representing different pathophysiological phenotypes of AKI has been proposed in human medicine [63]. The use of similar methods of classification might prove useful in veterinary medicine.

In this study, although we observed an association with negative prognostic indicators, neither uNGAL nor UNCR was associated with outcome. The absence of association with outcome could have been a result of the relatively small number of cases in this study leading to a difference being missed. A lack of power potentially leading to type 2 statistical error was further supported by the fact that even the previously identified negative prognostic indicators were not correlated with outcome in our cohort. Alternatively, it might be that the lack of association with outcome in our cohort was due to variability in the extent and magnitude of tubular injury; we saw a wide variation in the magnitude of increase in uNGAL and UNCR. There is some evidence to suggest that there is an association between outcome and the magnitude of increase in uNGAL; admission uNGAL concentration has been previously shown to be correlated with increased risk of in‐hospital mortality in human medicine [64, 65]. Although transfusion dependency is not an objective prognostic indicator, uNGAL was significantly different between dogs that required and those that did not require a transfusion in our cohort, which might indicate a higher uNGAL concentration is seen in more severely affected dogs, irrespective of outcome. Case stratification was not performed in this study due to the low number of cases and this could have been a reason for the absence of association between uNGAL and outcome.

We also need to recognize that our results could have been affected by factors such as the lack of standardization of treatment as well as the choice of euthanasia, which are often affected by factors other than disease severity, for example clinician preference and financial constraints, respectively. Given the correlation between uNGAL and UNCR and other negative prognostic indicators, these biomarkers should not be discounted as prognostic markers and warrant further analysis, especially in the context of multivariable analysis.

Our study has several limitations, including the inherent limitation relating to the specificity of NGAL as a marker of AKI. NGAL is released by neutrophils and epithelial cells of many different tissues, is then freely filtered through the glomerulus, and reabsorbed in the proximal tubule. Despite the high sensitivity of uNGAL to identify patients with AKI, its specificity has been questioned in both human and veterinary medicine as it originates from multiple tissues as well as from neutrophils and therefore might increase during inflammatory states [66, 67]. IMHA is an inflammatory disease process and therefore uNGAL above the reference range might reflect an abnormally high plasma NGAL. While there was no correlation between uNGAL/UNCR and leukocyte count, neutrophil count or CRP, we cannot exclude the possibility that high uNGAL levels merely reflected systemic inflammation. It is, however, interesting to note that, in a study of experimentally induced endotoxemia, uNGAL and UNCR were not significantly different to those of healthy dogs which would suggest that not all systemic inflammation will lead to high NGAL [43]. To overcome the issue of specificity, the use of plasma: uNGAL ratio has been proposed as an alternative to uNGAL for detection of AKI [68, 69].

Relative to NGAL's specificity, we recognize that previous data might suggest a mild correlation between hematuria and uNGAL concentrations. However, in the presence of conflicting evidence, this was not added as part of the exclusion criteria, especially considering that hemoglobinuria is considered to be a leading cause of renal injury in this cohort.

Another limitation of the study was the variable storage period of urine samples prior to analysis, which might have affected uNGAL levels. uNGAL is however stable at −80°C when stored for up to 5 years with insignificant decline in uNGAL levels over this period [70]. In a previous veterinary study evaluating uNGAL in AKI, the authors concluded that there was a trend towards a reduction in uNGAL with increasing duration of storage, but that this was not statistically significant [41]. Therefore, on balance, we think it is unlikely that storage will have significantly impacted on our results. As in the study by Nabity et al., we also observed high uNGAL values in some of the older samples.

One dog in our IMHA cohort presented with creatinine above the reference range and we are unable to be definitive about whether this reflected preexisting chronic kidney disease. While this dog had an uNGAL suggestive of AKI [34], it is possible that this was an acute‐on‐chronic renal injury, and that this affected not only uNGAL but also outcome. Also, the presence or suspicion presence of a thromboembolic event was retrospectively assessed which could have potentially led to misclassification of cases.

Lastly, due to ethical restrictions in the country that this study took place healthy dogs were not assessed for the presence of azotemia therefore we cannot definitively exclude the presence of subclinical renal disease. However, dogs were judged to be healthy as per our materials and methods including the absence of any clinical signs that might suggest renal disease.

In conclusion, we have demonstrated a significantly high uNGAL and UNCR in dogs with non‐associative IMHA. This is presumed to reflect acute renal tubular damage, raising the concern that AKI is underappreciated in this cohort of dogs. While uNGAL and UNCR were found to correlate with other negative prognostic indicators, in this study they did not appear to be individually associated with outcome.

## Ethics Statement

Approved by the University of Glasgow School of Veterinary Medicine Research Ethics Committee (reference number EA26/21). Authors declare human ethics approval was not needed for this study.

## Conflicts of Interest

The authors declare no conflicts of interest.

## Supporting information


Data S1.

## Endnotes

^1^
ADVIA 120, Siemens, Frimley, UK or in‐house ProCyte Dx Hematology Analyzer for hematology.

^2^
Dimension XPAND Siemens, Frimley, UK or in‐house Idexx Catalyst Dx for serum biochemistry.

^3^
Siemens Multistix 10SG Urinalysis Strips for urine dipstick.

^4^
Dimension XPAND, Siemens, Frimley, UK for urine creatinine.

^5^
Dog NGAL ELISA Kit, BIOPORTO Diagnostics, Hellerup, Denmark.

^6^
GraphPad Prism version 10.1.0 (316) for Windows, GraphPad Software, Boston, Massachusetts USA.

^7^
Dexadreson, MSD Animal Health, UK.

^8^
Prednicare, AnimalCare, UK.

^9^
Prednicare, AnimalCare, UK.

^10^
Cellcept, Roche, Switzerland.

^11^
Atopica, Elanco, USA.

^12^
Azathioprine, Boehringer Ingelheim, Roxane Laboratories, USA.

^13^
Intratect, Grifols UK Ltd., UK.

^14^
Clopidogrel, Sanofi, France.

^15^
Fragmin, Pfizer, UK.
