# Supplementary material for: Urine Neutrophil Gelatinase‐Associated Lipocalin in Non‐Associative Immune Mediated Hemolytic Anemia: A Prospective Controlled Study in 22 Dogs
Source: J Vet Intern Med. 2025 Jan 27;39(2):e70002. doi: 10.1111/jvim.70002 (PMC11772105; doi:10.1111/jvim.70002)
Supplement: Supplementary file 1 — Data S1. [file JVIM-39-e70002-s001.docx]

Supporting Information Table 1. Population characteristics and diagnosis of IMHA.

| Case | Breed | Age (years) | Sex | Slide agglutination | Spherocytosis | Direct Coombs | Hemoglobinuria | Hyperbilirubinemia (Tbil>0.58mg/dl) | ACVIM consensus statement diagnosis category |
| --- | --- | --- | --- | --- | --- | --- | --- | --- | --- |
| IMHA 01 | Cocker Spaniel | 8 | FN | Positive | Positive | Positive | Negative | yes | Diagnostic |
| IMHA 06 | Springer Spaniel | 10 | FN | Positive | Positive | n/a | Positive | yes | Diagnostic |
| IMHA 07 | Golden Retriever | 1 | FE | Positive | Negative | Positive | Negative | no | Supportive |
| IMHA 08 | Shit-tzu | 4 | FN | Positive | Positive | Positive | Negative | yes | Diagnostic |
| IMHA 09 | Cocker Spaniel | 4 | FE | Positive | Positive | n/a | Positive | yes | Diagnostic |
| IMHA 10 | Shit-tzu | 7 | FN | Positive | Positive | Positive | Positive | yes | Diagnostic |
| IMHA 13 | Schnauzer | 10 | FN | Negative | Positive | Positive | Negative | yes | Diagnostic |
| IMHA 16 | German Shepherd | 7 | ME | Negative | Equivocal | Positive | Negative | yes | Supportive |
| IMHA 20 | Crossbreed | 1 | FE | Positive | Positive | n/a | Positive | yes | Diagnostic |
| IMHA 21 | Cocker Spaniel | 12 | FN | Positive | Positive | Positive | Negative | no | Supportive |
| IMHA 002 | Labrador retriever | 11 | FN | Positive | Positive | Positive | Negative | yes | Diagnostic |
| IMHA 003 | Springer Spaniel | 10 | ME | Positive | Negative | Positive | Positive | yes | Diagnostic |
| IMHA 004 | Cocker Spaniel | 5 | FN | Positive | Equivocal | Positive | Negative | yes | Diagnostic |
| IMHA 005 | Whippet | 10 | FE | Positive | Positive | n/a | Positive | yes | Diagnostic |
| IMHA 007 | Cairn Terrier | 12 | MN | Positive | Positive | Positive | Negative | no | Supportive |
| IMHA 008 | Cocker Spaniel | 4 | FE | Positive | Positive | Positive | Positive | yes | Diagnostic |
| IMHA 009 | Irish Setter | 8 | ME | Positive | Positive | Positive | Positive | yes | Diagnostic |
| IMHA 013 | Schnauzer | 12 | FN | Positive | Positive | n/a | Negative | no | Supportive |
| IMHA 014 | Jack Russel Terrier | 9 | FN | Positive | Positive | Positive | Positive | yes | Diagnostic |
| IMHA 015 | Crossbreed | 9 | FN | Positive | Negative | Positive | Negative | no | Supportive |
| IMHA 020 | Cocker Spaniel | 6 | FN | Positive | Positive | n/a | Positive | yes | Diagnostic |
| IMHA 022 | Springer Spaniel | 5 | FN | Positive | Negative | Positive | Positive | yes | Diagnostic |

Abbreviations: IMHA: immune-mediated hemolytic anemia, FN: Female Neutered, FE: Female Entire, MN: Male Neutered, ME: Male Entire, n/a: not assessed, Tbil: Total bilirubin.

Supporting Information Table 2 Clinicopathological data of dogs with IMHA.

| Case | HCT  (%) | WBC  (x10^9/L) | Neutrophils  (x10^9/L) | Platelets  (x10^9/l) | Creatinine  (mg/dl) | Urea  (mg/dl) | Tbil  (mg/dl) | Albumin  (g/dl) | Total protein  (g/dl) | ALT  (U/l) | ALP  (U/l) |
| --- | --- | --- | --- | --- | --- | --- | --- | --- | --- | --- | --- |
| IMHA 01 | 4.7 | 48.9 | 28.67 | 182 | 0.44 | 44 | 1.58 | 2.8 | 6.3 | 44 | 172 |
| IMHA 06 | 9 | 23.8 | 18.7 | 412 | 0.87 | 109 | 3.04 | 3.4 | 7.2 | 706 | 119 |
| IMHA 07 | 21.8 | 17.03 | 9.22 | 134 | 0.75 | 32 | 0.41 | 3.3 | 6.8 | 14 | 43 |
| IMHA 08 | 11.8 | 26.65 | 16.92 | 197 | 0.74 | 51 | 2.90 | 3.2 | 7.2 | 41 | 375 |
| IMHA 09 | 19.7 | 19.9 | 14.27 | 735 | 0.54 | 34 | 1.64 | 3.2 | 8.5 | 20 | 85 |
| IMHA 10 | 9.9 | 18.46 | 13.107 | 350 | 0.62 | 47 | 1.64 | 3.8 | 7.2 | 38 | 197 |
| IMHA 13 | 14.2 | 8.1 | 6 | 81 | 0.60 | 45 | 1.23 | 2.7 | 5.2 | 38 | 57 |
| IMHA 16 | 13.6 | 16.9 | 12.675 | 142 | 0.98 | 39 | 1.23 | 2.9 | 6.6 | 29 | 79 |
| IMHA 20 | 20 | 13.36 | 10.688 | 209 | 0.79 | 56 | 0.82 | 2.6 | 6.7 | 25 | 175 |
| IMHA 21 | 16.6 | 18.4 | 14 | 146 | 0.32 | 25 | 0.41 | 3.6 | 7.9 | 52 | 160 |
| IMHA 002 | 19.6 | 14.44 | 10.32 | 190 | 1.06 | 30 | 1.11 | 3.6 | 7.2 | 26 | 58 |
| IMHA 003 | 11.6 | 22.94 | 17.664 | 125 | 0.67 | 73 | 8.36 | 2.8 | 6.9 | 25 | 133 |
| IMHA 004 | 25.5 | 9.62 | 7.05 | 125 | 0.58 | 47 | 3.45 | 3.7 | 7.6 | 36 | 185 |
| IMHA 005 | 19.9 | 35.25 | 24.675 | 401 | 0.72 | 35 | 0.94 | 2.2 | 6.5 | 30 | 251 |
| IMHA 007 | 17 | 19.02 | 15.106 | 269 | 0.72 | 53 | 0.41 | 3.3 | 7.3 | 89 | 482 |
| IMHA 008 | 13.2 | 26.78 | 16.87 | 153 | 0.40 | 95 | 20.58 | 4.2 | 9.3 | 103 | 76 |
| IMHA 009 | 10.3 | 51.15 | 29.667 | 285 | 2.86 | 162 | 39.01 | 3.4 | 7.2 | n/a | n/a |
| IMHA 013 | 22.9 | 13.67 | 10.936 | 333 | 0.54 | 37 | 0.23 | 3.4 | 8.2 | 72 | 578 |
| IMHA 014 | 19.5 | 15.92 | 13.691 | 350 | 0.67 | 49 | 1.05 | 2.7 | 6.6 | 1321 | 243 |
| IMHA 015 | 14.3 | 25.36 | 19.274 | 750 | 0.44 | 32 | 0.35 | 3.0 | 5.7 | 50 | 220 |
| IMHA 020 | 15.6 | 18.54 | 15.759 | 385 | 0.42 | 67 | 2.69 | 3.8 | 8.9 | n/a | 132 |
| IMHA 022 | 17 | 12.75 | 10.455 | 181 | 1.28 | 97 | 17.19 | 3.1 | 5.9 | 2704 | 360 |

Abbreviations: IMHA: immune-mediated hemolytic anemia, HCT: Hematocrit value, WBC: White blood cells, Tbil: Total bilirubin, ALT: Alanine transaminase, ALP: Alkaline phosphatase, n/a: not assessed.

Supporting Information Table 3. Urinalysis results.

|  | Case | USG | UPC | WBC/hpf (urine) | RBC/hpf (urine) | Hemoglobinuria | Glucosuria |
| --- | --- | --- | --- | --- | --- | --- | --- |
|  | IMHA 01 | 1.028 | 0.28 | negative | 0.5-1 | negative | negative |
|  | IMHA 06 | 1.022 | 3.88 | negative | 1-2 | Positive | negative |
|  | IMHA 07 | 1.022 | 0.11 | 0.5-1 | negative | negative | negative |
|  | IMHA 08 | 1.050 | 0.19 | negative | 0.5-1 | negative | trace |
|  | IMHA 09 | 1.038 | 11.57 | 3-4 | 3-4 | Positive | negative |
|  | IMHA 10 | 1.033 | 12.64 | 0.5-1 | 1-2 | Positive | negative |
|  | IMHA 13 | 1.032 | 0.05 | 0.5-1 | negative | negative | negative |
|  | IMHA 16 | 1.030 | 0.12 | 1-2 | 0.5-1 | negative | negative |
|  | IMHA 20 | 1.050 | 5.41 | 0.5-1 | 0.5-1 | positive | negative |
|  | IMHA 21 | 1.024 | 0.29 | 0.5-1 | 0.5-1 | negative | negative |
|  | IMHA 002 | 1.032 | 0.15 | 3-4 | negative | negative | negative |
|  | IMHA 003 | 1.024 | 3.43 | 1-2 | negative | positive | negative |
|  | IMHA 004 | 1.034 | 0.39 | negative | negative | negative | negative |
|  | IMHA 005 | 1.018 | 0.76 | 0.5-1 | negative | positive | negative |
|  | IMHA 007 | 1.042 | 0.18 | negative | negative | negative | negative |
|  | IMHA 008 | 1.018 | 16.5 | 0.5-1 | 0.5-1 | positive | negative |
|  | IMHA 009 | 1.050 | excl | negative | 3-4 | positive | negative |
|  | IMHA 013 | 1.017 | 6.17 | negative | negative | negative | negative |
|  | IMHA 014 | 1.030 | 0.97 | negative | negative | positive | negative |
|  | IMHA 015 | 1.028 | 0.61 | negative | negative | negative | negative |
|  | IMHA 020 | 1.030 | 6.26 | negative | negative | positive | negative |
|  | IMHA 022 | 1.038 | 3.32 | 0.5-1 | 0.5-1 | positive | negative |

Abbreviations: IMHA: immune-mediated hemolytic anemia, USG: Urine specific gravity, UPC: Urine protein to creatinine ratio, WBC: White blood cells, RBC: Red blood cells, hpf: high power field, excl: excluded.

Supporting Information Table 4. Treatment information.

|  |  |  | |  |  |  |  |  | | | |  |  |  |  |
| --- | --- | --- | --- | --- | --- | --- | --- | --- | --- | --- | --- | --- | --- | --- | --- |
| Case | | | Treatment  (IS 1) | | Dose (mg/kg/day)  (IS1) | Treatment  (IS 2) | Dose (mg/kg/day)  (IS2) | Treatment  (AT 1) | Dose (mg/kg/day)  (AT 1) | Treatment (AT 2) | Dose (units/kg 3 times/day) (AT 2) | | Additional  IS treatment | Transfusion? | No of transfusions |
| IMHA 01 | | | dexamethasone | | 0.3 | mycophenolate | 10 | clopidogrel | 2.7 |  |  | |  | yes | 2 |
| IMHA 06 | | | dexamethasone | | 0.3 | mycophenolate | 20 | clopidogrel | 2.2 |  |  | |  | yes | 1 |
| IMHA 07 | | | dexamethasone | | 0.3 | mycophenolate | 19.2 | clopidogrel | 2.1 |  |  | |  | no | 0 |
| IMHA 08 | | | dexamethasone | | 0.3 | mycophenolate | 12 | clopidogrel | 2.2 |  |  | |  | yes | 2 |
| IMHA 09 | | | dexamethasone | | 0.3 | azathioprine | 1.6 | clopidogrel | 2.38 |  |  | |  | yes | 1 |
| IMHA 10 | | | dexamethasone | | 0.3 | mycophenolate | 20 | clopidogrel | 2 |  |  | |  | yes | 1 |
| IMHA 13 | | | dexamethasone | | 0.3 | mycophenolate | 19 | clopidogrel | 1.6 |  |  | |  | yes | 2 |
| IMHA 16 | | | dexamethasone | | 0.3 | mycophenolate | 20 | clopidogrel | 2 |  |  | |  | yes | 2 |
| IMHA 20 | | | dexamethasone | | 0.3 | mycophenolate | 21.2 | clopidogrel | 3.2 |  |  | | Human IVIG | yes | 3 |
| IMHA 21 | | | dexamethasone | | 0.3 |  |  | clopidogrel | 3 | dalteparin | 150 | |  | yes | 1 |
| IMHA 002 | | | dexamethasone | | 0.31 | mycophenolate | 7.9 | clopidogrel | 2.4 |  |  | |  | no | 0 |
| IMHA 003 | | | dexamethasone | | 0.3 | mycophenolate | 10 | clopidogrel | 2.8 |  |  | | Human IVIG | yes | 2 |
| IMHA 004 | | | prednisolone | | 1.94 | mycophenolate | 13.9 | clopidogrel | 2.1 |  |  | |  | yes | 2 |
| IMHA 005 | | | prednisolone | | 2 | cyclosporine | 5 | clopidogrel | 3.8 |  |  | |  | yes | 1 |
| IMHA 007 | | | dexamethasone | | 0.3 |  |  | clopidogrel | 1.7 |  |  | |  | no | 0 |
| IMHA 008 | | | dexamethasone | | 0.3 | mycophenolate | 10 | clopidogrel | 1.6 |  |  | |  | yes | 2 |
| IMHA 009 | | | dexamethasone | | 0.3 | mycophenolate | 10 | clopidogrel | 1.2 |  |  | |  | yes | 1 |
| IMHA 013 | | | prednisolone | | 2.02 | mycophenolate | 20.2 | clopidogrel | 1.9 |  |  | |  | no | 0 |
| IMHA 014 | | | dexamethasone | | 0.3 | cyclosporine | 7.8 | clopidogrel | 2 |  |  | |  | yes | 2 |
| IMHA 015 | | | dexamethasone | | 0.07 | mycophenolate | 24.2 | clopidogrel | 2.8 |  |  | |  | yes | 1 |
| IMHA 020 | | | dexamethasone | | 0.31 | mycophenolate | 13 | clopidogrel | 1 |  |  | |  | yes | 2 |
| IMHA 022 | | | dexamethasone | | 0.6 | mycophenolate | 19.5 | clopidogrel | 1.8 |  |  | |  | yes | 1 |

Abbreviations: IMHA: immune-mediated hemolytic anemia, IS: Immunosuppression, AT: antithrombotics, Human IVIG: Human immunoglobulin.

Supporting Information Table 5. Outcome data.

| Case | CHAOS score | ASA score | Dead at discharge | Died after discharge | Time of death (days) | Cause of death | Follow-up (days) | Suspicion of TE |
| --- | --- | --- | --- | --- | --- | --- | --- | --- |
| IMHA 01 | 4 | 4 | no | no |  |  | 180 | yes |
| IMHA 06 | 3 | 4 | no | no |  |  | 240 | no |
| IMHA 07 | 1 | 2 | no | no |  |  | 20 | no |
| IMHA 08 | 1 | 4 | no | no |  |  | 180 | no |
| IMHA 09 | 2 | 4 | no | no |  |  | 7 | no |
| IMHA 10 | 4 | 4 | no | no |  |  | 180 | yes |
| IMHA 13 | 3 | 3 | yes | n/a | 6 | persistent hemolysis |  | no |
| IMHA 16 | 3 | 4 | no | no |  |  | 30 | no |
| IMHA 20 | 3 | 2 | yes | n/a | 7 | persistent hemolysis |  | no |
| IMHA 21 | 3 | 4 | yes | n/a | 2 | suspected CNS thrombosis |  | yes |
| IMHA 002 | 4 | 3 | no | yes | 319 | unrelated |  | no |
| IMHA 003 | 6 | 4 | no | yes | 58 | IMHA relapse |  | yes |
| IMHA 004 | 1 | 3 | no | no |  |  | 654 | no |
| IMHA 005 | 4 | 3 | no | no |  |  | 732 | no |
| IMHA 007 | 3 | 2 | no | yes | 473 | unrelated |  | no |
| IMHA 008 | 4 | 4 | yes | n/a | 6 | renal tubular acidosis |  | no |
| IMHA 009 | 6 | 4 | yes | n/a | 2 | AKI |  | no |
| IMHA 013 | 3 | 3 | no | no |  |  | 319 | no |
| IMHA 014 | 4 | 4 | yes | n/a | 6 | persistent hemolysis |  | no |
| IMHA 015 | 3 | 3 | no | yes | 68 | unknown |  | no |
| IMHA 020 | 1 | 3 | no | no |  |  | 1,080 | no |
| IMHA 022 | 3 | 4 | yes | n/a | 1 | suspected CNS thrombosis |  | yes |

Abbreviations: IMHA: immune-mediated hemolytic anemia, CHAOS: Canine hemolytic anemia objective score, ASA: American Society of Anesthesiologists, TE: Thromboembolism, n/a: not assessed, CNS: Central nervous system, AKI: Acute kidney injury.
